# Production of xylitol and bio-detoxification of cocoa pod husk hemicellulose hydrolysate by *Candida boidinii* XM02G

**DOI:** 10.1371/journal.pone.0195206

**Published:** 2018-04-11

**Authors:** Nivio Batista Santana, João Carlos Teixeira Dias, Rachel Passos Rezende, Marcelo Franco, Larissa Karen Silva Oliveira, Lucas Oliveira Souza

**Affiliations:** 1 Department of Animal and Rural Technology, State University of Southwest Bahia (UESB), Itapetinga, Bahia, Brazil; 2 Department of Exact and Technological Sciences, State University of Santa Cruz (UESC), Ilhéus, Bahia, Brazil; 3 Department of Biological Sciences, State University of Santa Cruz (UESC), Ilhéus, Bahia, Brazil; 4 Biomedicine, State University of Santa Cruz, Ilhéus, Bahia, Brazil; 5 Postgraduate Program in Food Science and Engineering, State University of Southwest Bahia (UESB), Itapetinga, Bahia, Brazil; Babasaheb Bhimrao Ambedkar University, INDIA

## Abstract

The use of cocoa pod husk hemicellulose hydrolysate (CPHHH) was evaluated for the production of xylitol by *Candida boidinii* XM02G yeast isolated from soil of cocoa-growing areas and decaying bark, as an alternative means of reusing this type of waste. Xylitol was obtained in concentrations of 11.34 g.L^-1^, corresponding to a yield (Y_p/s_) of 0.52 g.g^-1^ with a fermentation efficiency (ε) of 56.6%. The yeast was tolerant to inhibitor compounds present in CPHHH without detoxification in different concentration factors, and was able to tolerate phenolic compounds at approximately 6 g.L^-1^. The yeast was also able to metabolize more than 99% (p/v) of furfural and hydroxymethylfurfural present in the non-detoxified CPHHH without extension of the cell-growth lag phase, showing the potential of this microorganism for the production of xylitol. The fermentation of cocoa pod husk hydrolysates appears to provide an alternative use which may reduce the impact generated by incorrect disposal of this waste.

## Introduction

Xylitol is used as a sweetener in the food, pharmaceutical and personal care industries, and its commercial production is currently limited to chemical synthesis by catalytic hydrogenation of xylose extracted from vegetable fibers [1,2]. Benefits of xylitol in terms of human health make it a product of great value and interest in various lines of research. Its organic synthesis by microorganisms has been studied with the aim of reducing the disadvantages presented by chemically reducing the xylose purification steps and removing waste from the catalyst. Various types of agro-industrial waste have been studied as a potential source of substrate for fermentation and generation of this compound [3–6].

However, the biotransformation of xylose into xylitol using yeast requires the production of hydrolysates from the residues, which are rich in sugars, and it is thus required to reduce their toxicity, which occurs due to the presence of phenolic compounds derived from the breakdown of lignin, the presence of furans and organic acids, which in turn reduce the fermentability of the medium to inhibit the microbial metabolism [7,8]. Purification processes have been proposed and evaluated to enable the use of hydrolysates, but these processes can reduce the concentration of available sugars and raise the cost of the operation due to the chemicals that are required for the removal of unwanted molecules. An alternative to the chemical process is the use of microorganisms which are able to metabolize these inhibitor compounds, transforming the substrates present without the need for purification. Microorganisms with high tolerance to inhibitory compounds are of great interest because they can reduce costs and eliminate certain steps in the process [9–13].

Cocoa is the main crop grown in southern Bahia; it has singular importance in the local economy, as well as in the national and global production of chocolate. Nevertheless, the extraction of the beans which are destined for the production of chocolate generates large amounts of waste, consisting mainly of husk, which in turn represents approximately 80% of the fruit by weight [14].

World cocoa beans production in the 2015–2016 harvest was 4,013 million tons [15], generating around 16 million tons of cocoa pod husks. Discarded lignocellulosic material can be used to generate energy and to produce chemicals for various industrial sectors [7], however, cocoa pod husks are rarely reused. Alternatives to this material have been proposed primarily in the field of animal nutrition, agriculture and chemistry [15–26]. Notwithstanding, studies on its use in fermentation processes remain scarce [18].

Considering the above, the present study investigates the use of cocoa pod husks as a source of xylose for the production of xylitol using the *Candida boidinii* XM02G yeast, which has a high tolerance to inhibitor compounds, isolated from soils in the cocoa cultivation area and from the decaying husks.

## Materials and methods

### Characterization of waste

The residues were donated by small traders of cocoa-based products in the southern area of Bahia. The residues were washed with potable water, dried in an oven at 70°C until 3% humidity was achieved, ground and sieved (30 mesh). The content of fiber, protein, ether extract and dry residue matter were determined in accordance with the methodology of the Association of Official Analytical Chemists [27].

### Microorganism and medium preparation

*C*. *boidinii* was isolated from the soil and decomposing cocoa pod husks in cocoa cultivation areas of the campus of the State University of Santa Cruz *(14° 47' 55.50" S 39° 10' 20.03" W)*, in the city of Ilhéus, state of Bahia. No permissions were required for collections in the campus area. This study did not involve endangered species. DNA extraction was done according to Silva-Filho et al [28]. Amplification of the D1 / D2 divergent domains of the 26S rDNA subunit was done using the primers NL1-(5`GCCATATCAATAAGCGGAGG 3`) and NL4-(5`GGTCCGTGTTTCAAGACGG 3`). The sequence obtained (TCAAGACGGGCGGTATTAGACCATTACGCCAGCATCCTAGGCAAAGCCGCAGACCTCAGTCTAGATAGGCAGTATCAACTGAAGCTATAACACTCCGAAGAGCCACGTTCAACAGTTCTTATCCTGCCACCTAAACTGATGCTGGCCCAATAAAAAGCTAGAGCACCATCCACAAGGAACAGTGTTAACTGAATATCAGTCTGATCAAATACCCTTCCCTTTCAACAATTTCACGTACTTTTTCACTCTCTTTTCAAAGTTCTTTTCATCTTTCCATCACTGACTTGTTCGCTATCGGTCTCTCGCCAATATTTAGCTTTAGATGGAATTTACCACCCACTTAGAGCTGCATTCCCAAACAACTCGACTCGTCRAAAGTATCTTACATAGAATGGGCACCCCATCGCACGGGATTCTCACCCTCTGTGACGTCCTGTTCCAAGGAACATAGACAAGGGCCAACTCCAAGATTACTTTCTTCAAATTACAACTCGGATACTGAAAGTACCAGATTTCAAATTTGAGCTCTTGCCGCTTCACTCGCCGCTACTAAGGCAATCCCTGTTGGTTTCTTTTCCTCCGCTTATTGATATGC) was compared with the sequences available in Genbank (Accession No. KY106341), showing to be 99% similarity with *C*. *boidinii* XM02G. The microorganism was kept in Sabouraud agar slants covered with mineral oil, in refrigerated conditions. YPX medium composed of yeast extract (5 g.L^-1^), peptone (5 g.L^-1^), xylose (20 g.L^-1^) [12] was used for the reactivation of the culture. Cell growth was determined by spectrophotometry at a wavelength of 600 nm and correlated to the dry weight of the cells (g.L^-1^) using standard curve [27].

### Experimental design and statistical analysis on obtaining the hemicellulose hydrolysate

Optimization of the process for obtaining CPHHH was evaluated by means of a Central Composite Rotational Design (CCRD) 2^2^ experimental design, with 4 different experiments, 4 axial points and 3 central points. The corresponding matrix is shown in Table 1. The analyzed independent variables (factors) and their ranges were sulfuric acid concentration and time of pretreatment; the analyzed dependent variable (response) was the concentration of xylose. All experiments were conducted at 120°C and 1.0 atm. The proportion between husk mass and the volume of acid solution was 1/8 (p/v). The hydrolysates were centrifuged at 2000 *g* for 10 mins to remove the non-hydrolyzed fraction. The supernatant was filtered using a 0.22 μm PES filter with 13mm diameter and was retained for subsequent analysis. The CPHHH was evaporated at 70°C in a vacuum oven until the concentrations that were 2-, 3- and 4 (2×, 3× and 4×) times the original concentration of the hydrolysates. The data obtained were analyzed with the aid of STATISTICA® software v.10.0 (Statsoft, USA).

**Table 1:** Experimental design for the optimization experiment of cocoa pod husks hydrolysis.

Treatment	Time (*X*_*1*_*-*min)	Acid (*X*_*2*_*-* %v/v)
1	-1 (21,6)	-1 (1,01)
2	+1 (78,4)	-1 (1,01)
3	-1 (21,6)	+1 (3,49)
4	+1 (78,4)	+1(3,49)
5	0 (50)	0 (2,25)
6	0 (50)	0 (2,25)
7	0 (50)	0 (2,25)
8	-1,41 (10)	0 (2,25)
9	+1,41 (90)	0 (2,25)
10	0 (50)	-1,41 (0,5)
11	0 (50)	+1,41 (4)

### Hydrolysate detoxification

#### pH adjustment and adsorption with activated charcoal

Calcium hydroxide [Ca(OH)_2_] was added to the CPHHH until a pH 10 was achieved. After remaining at that pH for 1 h, the hydrolysate was centrifuged at 2000 *g* for 10 min, acidified to pH 5.5 with H_3_PO_4_ and centrifuged once more after 1 h. Activated charcoal was added to the hydrolysate at a rate of 2% (w/v) at 30°C, stirred at 200rpm for 1 h and then centrifuged 2000 *g* [5].

#### Use of ion-exchange resins

The pH of the hydrolysate was previously adjusted to 5 using Ca(OH)_2_ and centrifuged at 2000*g*to remove the precipitate. The ion-exchange resins, Amberlit IRA 120 and Amberlit IRA 410, were hydrated in distilled water for 24 h. The CPHHH was treated with Amberlit IRA 410 and stirred at 200 rpm for 30 min at 30°C. After this process, the hydrolysate was drained and treated with Arberlit IRA 120 and stirred at 200 rpm for 30 min at 30°C. The ratio of hydrolysate volume to resin mass was 2:1 [28].

### Fermentation of the CPHHH

#### Fermentation of the CPHHH without detoxification by *C*. *boidinii*

The inoculum for cultivation in liquid medium was produced in hydrolysate enriched with yeast extract (5 g.L^-1^), peptone (5 g.L^-1^), ammonium sulphate (2 g.L^-1^), dibasic potassium phosphate (2 g.L^-1^) and hydrated hepta magnesium sulphate (1 g.L^-1^). Cells adapted in petri dishes were removed and transferred to the liquid medium at the original concentration. After 96 h, 5 ml aliquots were removed and re-inoculated into the more concentrated medium (2×). This procedure was repeated until the highest concentrations of hydrolyzate (3× and 4×). The initial cell concentration in fermentations was 0.05 g.L^-1^.

#### Fermentation of the physicochemically-detoxified CPHHH

The CPHHH detoxified by pH adjustment and activated charcoal was concentrated until it reached four times the original concentration. The inoculum was prepared based on a 48-h culture in YPX medium. Next, the adaptation of the inoculum was carried out in the CPHHH at the original concentration for 48 h and then transferred to the CPHHH with doubled concentration (2×) and to CPHHH (4×) for 48 h each. The medium was centrifuged at 5000 *g* and the cells pellet was then resuspended at 4 g.L^-1^ in the CPHHH enriched with yeast extract (5 g.L^-1^), peptone (5 g.L^-1^), ammonium sulphate (2 g.L^-1^), dibasic potassium phosphate (2 g.L^-1^) and magnesium sulfate heptahydrated (1 g.L^-1^). Fermentation was carried out in 125-mL Erlenmeyer flasks containing 50 mL of hydrolysate and stirred at 110 rpm.

### Analytical methods

The content of sugars and xylitol were determined by high performance liquid chromatography (HPLC), using a Shimadzu system equipped with refractive index detector, Shimpack CLC-NH_2_ column and acetonitrile/water (80/20) as the mobile phase at 1.0 mL.min^-1^(30°C). Furfural and hydroxymethylfurfural (HMF) were quantified by high performance liquid chromatography (HPLC) using a Shimadzu system equipped with CLC-ODS column, kept at 40°C and with detector diode arrangement at a wavelength of 280 nm. The mobile phase was initially composed of water/methanol (95/5 p/v), andreached 100% of methanol after 13 min at a flow rate of 0.8 mL.min^-1^.

Total phenolic compound content was determined using the Folin Ciocalteau reagent and expressed as g.L^-1^ of ferulic acid [29].

## Results and discussion

### Optimization of the acid hydrolysis of cocoa residue

**Fig 1:**
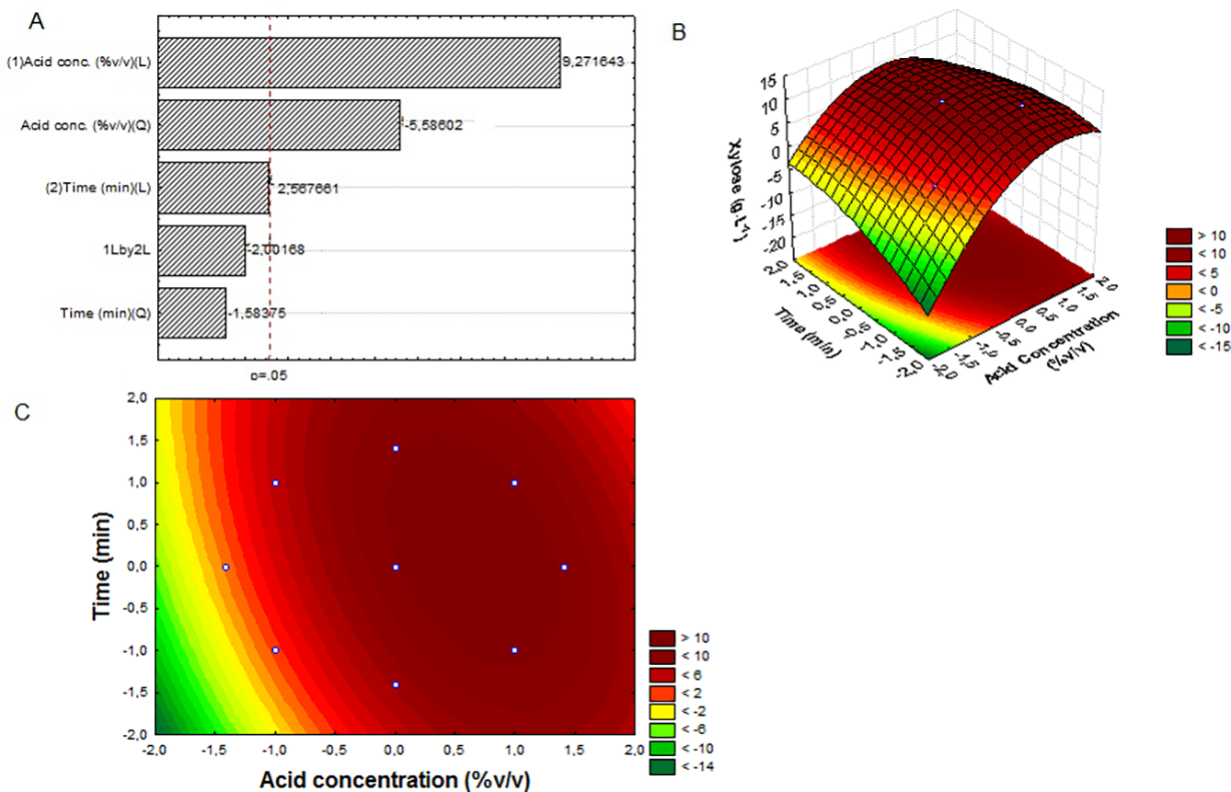
Pareto chart of the data analysis (p<0.05); (A) surface response generated by the model adjusted for the extraction of hemicellulose from cocoa pod husks in three-dimensional (B) and two-dimensional (C) shape.

The Pareto chart (Fig 1A) shows the significance of the variables tested at the level of probability of *p*<0.05. It was observed that the effect of heating time on the results was not significant, while the concentration of the acid had a considerable influence on hydrolysis. Long hydrolysis times favored the conversion of sugars into products such as furfural and HMF, thus reducing the concentration of substrate available for fermentation. Nevertheless, depending on the type of acid used in the hydrolysis and the pressure applied during heating, formation of furans can also occur [30,31]. This may explain why the time factor was found not to be meaningful. The model containing the linear and quadratic factors of the acid concentration is shown below, displaying the regression coefficient (R^2^) of 0.93216 and a non-significant lack of adjustment (*p* = 0.56):

$$xylose\;({g.L}^{-1})=9.90656+3.38123{\;X}_{1}-2.43084{\;X}_{1}^{2}$$ (1)

Where *X*_*1*_ represents the encoded variable corresponding to the concentration of the acid.

Fig 1B and 1C show the response surface generated from the model obtained from the data analysis. It is possible to observe that two experimental points are within the highest-concentration range obtained for xylose. The critical point was 0.19 for time (*X*_*1*_) and 0.65 for the acid (*X*_*2*_), with a predicted maximum value of 11.03 g.L^-1^ of xylose for the response variable “concentration of xylose”. Therefore, the authors have selected the combination +1/-1 for the encoded variables as the most suitable for the available work conditions. These include 3.49% acid concentration and 21.6 min length of stay under heating. The concentration of xylose obtained in these coordinates was 11 g.L^-1^. There are published reports of xylose concentrations of 15.7 g.L^-1^ for sugarcane bagasse, 45.6 g.L^-1^ for barley and bran, and 19.21 g.L^-1^ for eucalyptus, among other raw materials [32].

Table 2 shows the composition of the cocoa pods used in the experiment before and after the hemicellulose extraction process. The acid hydrolysis process removed 95.7% of the hemicellulose present in the husk. Consequently, the remaining residues increased proportionally in their concentrated fractions after the hydrolysis. Other authors have reported similar composition for cellulose, lignin and protein in the cocoa pod husks. However, the values reported for hemicellulose by other authors are less than those observed in the present study [14, 24, 25]. According to these authors, the predominant component of the husks included lignocellulosic material (32 to 45%) and pectin, with low levels of crude protein (6 to 9%) and total sugars (3%) expressed as a percentage of dry matter. In the present study, no soluble sugars were observed in the chromatographic determination of the water extract of the husks.

**Table 2:** Composition of cocoa pod husks residue before and after extraction of hemicelluloses.

Parameter(%)	Cocoa Pod Husk	Residue from hydrolysis
Dry matter	89,8	91,2
ADF*	56,34	87,03
NDF**	77,43	87,92
Hemicellulose	21,09	0,89
Cellulose	30,79	43,15
Lignin	25,55	43,88
Ethereal extract	0,68	1,00
Protein	6,93	6,62

* Acid Detergent Fiber

**Neutral Detergent Fiber

### Characterization of the hydrolysate

Xylose (7.24 g.L^-1^), glucose (4.55 g.L^-1^) and arabinose (2.64 g.L^-1^) were produced during the hydrolysis process (NAT treatment). According to Table 3, it is possible to observe that, in addition to xylose, glucose was produced in large proportion and arabinose in smaller proportions. The composition of sugars in the biomass hydrolysates depends on the composition of the raw material and the intensity of the treatment [7]. Generally, hemicellulose hydrolysates obtained from soft hydrothermal treatments have a higher proportion of xylose compared to glucose [10–12]. The lower concentrations of acid investigated in this study did not produce satisfactory xylose extraction results (Fig 1B). The relatively high acid concentration in the chosen treatment may partly explain the high concentration of glucose, which was caused by hydrolysis of the cellulose in the cocoa pods. The high glucose concentration may also be related to the large amount of mucilage present in the inner part of the husk, which is rich in sugars such as glucose and fructose, in addition to pectin which was found in considerable quantities and which may have contributed to the generation of glucose and other sugars. Salgado et al. obtained concentrations of 10.7 g.L^-1^for glucose, 14.7 g.L^-1^for xylose and 1.6 g.L^-1^for arabinose from vineyard residues [33]. Rocha et al. reported a glucose concentration of 17.8 g.L^-1^ and a xilarabina concentration of 34.0 g.L^-1^ in cashew residue hydrolysates [34]. With respect to the concentration of total phenolics, furfural and HMF, the values obtained for the original hydrolysate (NAT) are similar to those obtained in other sources of biomass [12], which shows that there was no excessive degradation of sugars and lignin caused by the heating time or the acid concentration used in the process developed in the present study.

**Table 3:** Characterization of the hydrolyzates according to the treatments applied for detoxification.

Trat.	Xil (g.L^-1^)	Ara (g.L^-1^)	Glu (g.L^-1^)	FUR(g.L^-1^)	HMF(g.L^-1^)	TPC (g.L^-1^)
NAT	7.24 ^C^ (±1.16)	2.64 ^B^ (±0.86)	4.55^B^ (±1.03)	0.33^A^ (±0.20)	0.41^A^ (±7 E^-4^)	0.91^A^ (±0.17)
PH 5	7.91 ^C^ (±1.17)	3.57 ^B^ (±0.80)	5.59^B^ (±0.67)	0.19^B^ (±0.06)	0.22^B^(±0.10)	0.99^A^ (±0.07)
AMB 410	8.00 ^C^ (±0.73)	1.54 ^B^ (±0.31)	4.66^B^ (±0.09)	0.084^B^ (±0.002)	0.13^B.C^ (±0.11)	0.27^C.D^ (±0.04)
AMB 120	8.43 ^C^ (±0.34)	n.d	2.93^B^ (±0.85)	0.03^B^ (±0.004)	0.04^B.C^ (±0.018)	0.01^D^ (±0.01)
PH 10	5.22 ^C^ (±1.73)	2.43 ^B^ (±0.93)	3.25^B^ (±1.01)	0.026^B^ (±0.023)	0.025^B.C^ (±0.02)	0.52^B.C^ (±0.08)
PH 5.5	7.66^C^ (±1.04)	2.58 ^B^ (±1.11)	4.93^B^ (±0.47)	0.049^B^ (±0.033)	0.083^B.C^ (±0.09)	0.70^A.B^ (±0.12)
DETOX 1×	7.93^C^ (±2.51)	1.83 ^B^ (±0.55)	6.24^B^ (±0.85)	0.004^B^ (±6 E^-4^)	0.003^C^ (±7E^-4^)	0.15^D^ (±0.05)
DETOX 2×	15.37^B^ (±4.81)	2.31^B^(± 4.0)	12.09^B^ (±1.00)	0.003^B^ (±0.003)	0.007^C^(±0.006)	0.29^C.D^ (±0.10)
DETOX 4×	33.13^A^ (±3.86)	11.40^A^ ±0.15)	26.20^A^(±5.70)	0.01^B^ (±0.002)	0.015^C^ (±0.004)	0.53^B.C^ (±0.15)

Xyl: xylose; Ara: arabinose; Glu: glucose; FUR: furfural; HMF: Hydroxymetilfurfural; TPC: Total phenolic compounds

NAT: Hydrolyzate obtained without any treatment

pH 5: Hydrolyzate with pH adjusted to 5, used for treatment with resins.

AMB 410 e AMB 120: Treated hydrolyzate after the use of ion exchange resin Ambertlit IR 410 and Amberlit IR 120, respectively.

pH 10 e pH 5,5: Hydrolyzate composition after each pH adjustment.

Detox 1×, 2×, 4×: Detoxified hydrolyzate after pH adjustment and activated charcoal, without concentrating and concentrating 2 and 4 times.

n.d: not detected.

Means followed by the same letter (A,B,C or D) in the same column do not differ by Tukey's test (p<0,05).

### Detoxification processes

#### Detoxification by pH adjustment and adsorption with activated charcoal

After adjusting the pH value to 10 and then to 5.5, there was no significant loss of xylose. However, these steps do not result in the removal of phenolic compounds. Huang et al. reported that alkalization lead to loss of sugars, which causes a reduction in fermentation efficiency, in addition to requiring additional processes for the removal of precipitated calcium sulfate or calcium phosphate [12]. After the application of activated charcoal (DETOX), there was a 78% decrease in phenolic compounds and the concentration remained at 0.15 g.L^-1^, showing that this step is fundamental to the process of detoxification of CPHHH. Misra et al. noted that several reports suggest that the presence of activated charcoal in the hydrolysate helps primarily with the reduction of phenolic compounds generated during acid hydrolysis [3]. Ge et al. reported that alkalization and activated charcoal treatment resulted in the complete removal of furfural, while acetic acid and phenolic compounds decreased by 62.4% and 96.6%, respectively [35]. In the present study, the reduction in concentration of furfural and HMF by means of the same method was greater than 99%.

#### Use of ion-exchange resins

The resin treatment almost completely eliminated the total phenolic compounds, furfural and HMF, as well as the arabinose present in the compounds. There was no significant loss of xylose or glucose after treatment with ion-exchange resins. Canilha et al. reported that there was less loss of sugars with resin treatment compared to pH adjustment and use of activated charcoal [36]. There were no significant differences between the methodologies used for detoxification in the present study.

### Fermentation of the CPHHH

#### Fermentation of the CPHHH without detoxification by *C*. *boidinii*

**Fig 2:**
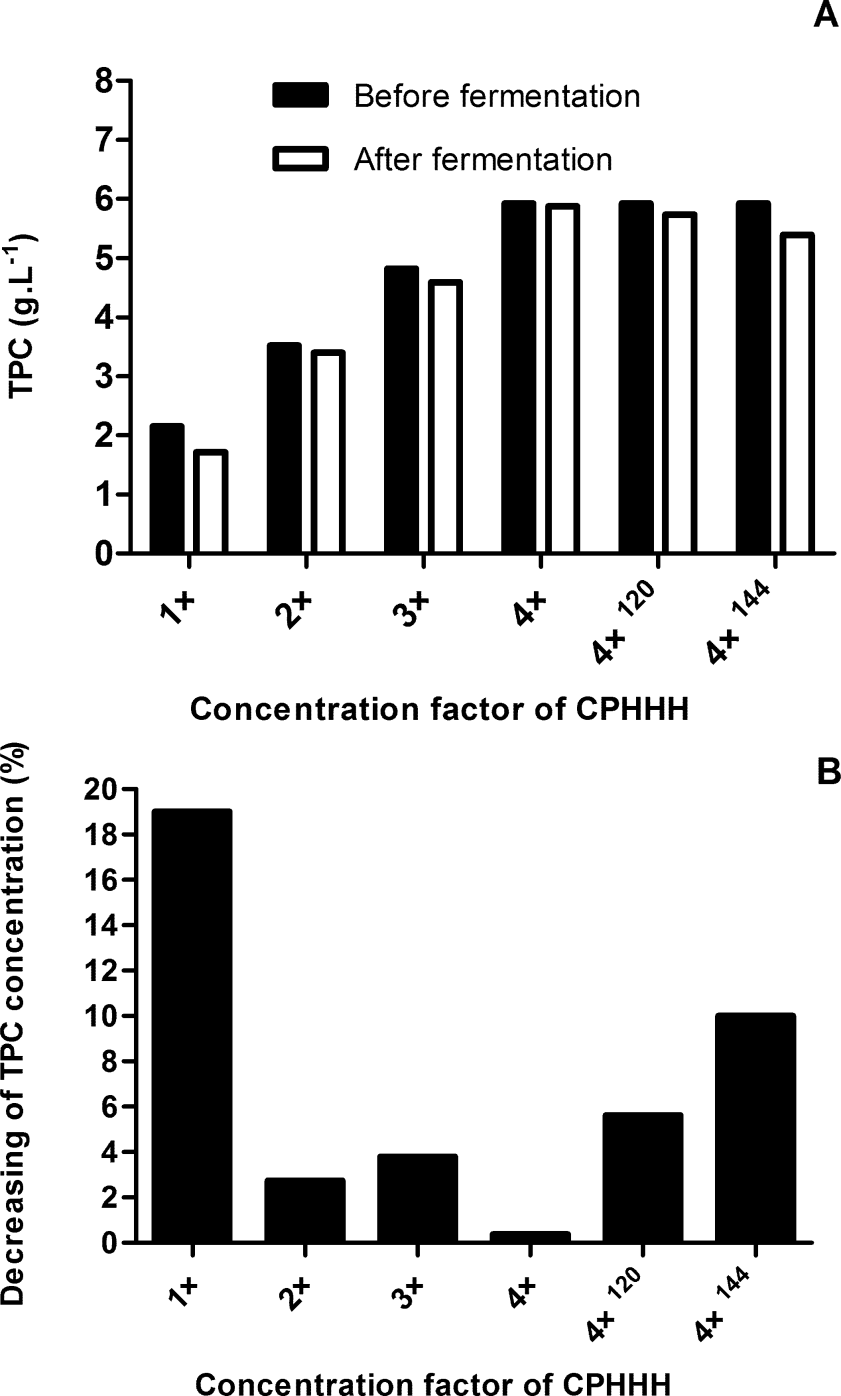
Concentration of TPC (g.L^-1^) in the CPHHH before (black columns) and after (white columns) the growth of *C*. *boidinii* (A) and percentage of TPC decreasing after fermentation (B).

Despite the high concentration of inhibitors, it was possible to observe cell growth at all concentrations of the hydrolysate in liquid medium composed of non-detoxified CPHHH. The initial concentrations of total phenolics compounds in the non-detoxified CPHHH (original and concentrated 2×, 3× and 4×) were 2.15, 3.52, 4.82 and 5.92 g.L^-1^, respectively. Despite the low inoculation rates used in this study (approximately 0.05 g.L^-1^), *C*. *boidinii* was able to reduce the concentration of total phenolic compounds at all levels in the medium (Fig 2A).

Although the greatest reduction (19.01%) was observed in the medium with the lowest (original) concentration after 96 h, the highest phenolic concentration present with other treatments did not cause complete inhibition of cellular activity. Even with the medium that was concentrated four times, it is possible to note the continued reduction of TPC after 120 h and 144 h of cultivation, for which the second highest percentage reduction of these compounds was observed (Fig 2B). All of these data show the adaptability of the isolate of *C*. *boidinii* to conditions that are considered aggressive for yeast [37,38]. Nevertheless, the values for biological reduction of phenolic compounds were lower than those obtained for the alkali treatment and activated charcoal, and with the use of ion-exchange resins (Table 3), which show removal rates varying between 78% and 100%.

Table 4 shows the consumption of substrate and cell growth for the treatments tested in this study. It is noted that the increase in concentration of CPHHH resulted in lower consumption of substrate and cell growth during the growing period that was investigated. However, none of the conditions tested was aggressive enough to cause death or total inhibition of the culture, showing the high tolerance of the *C*. *boidinii* isolated in supporting high concentrations of inhibitor compounds. Despite the low consumption of xylose in the treatment with the highest concentration factor for CPHHH, microbial activity could be demonstrated by the significant consumption of glucose. With regard to the conversion factor for the cell substrate (Y_x/s_), CPHHH 1× was the condition that produced the highest value for this parameter.

**Table 4:** Xylose and glucose consumption and biomass formation by *C*. *boidinnii* in CPHHH without detoxification.

Concent. factor	Xil_i_(g.L^-1^)	Xil_f_ (g.L^-1^)	Cons.Xil. (%)	Gli_i_ (g.L^-1^)	Gli_f_ (g.L^-1^)	Cons. Gli. (%)	X_f_(g.L^-1^)	Y_x/s_(g.g^-1^)
1×	7.37	0.54	92.72	5.50	0.43	92.15	5.25	0.43
2×	12.02	4.60	61.70	8.15	0	100	3.83	0.24
3×	17.52	11.39	34.98	12.78	0	100	2.95	0.15
4×	23.81	23.81	0	17.67	9.95	43.70	2.53	0.33
4×_120_	23.81	21.32	10	17.67	0	100	2.77	0.14
4×_144_	23.81	21.32	10	17.67	0	100	3.22	0.16

Xil_i_ e Xil_f_: initial and final concentrations of xylose respectively

Gli_i_ e Gli_f_: initial and final glucose concentrations respectively

Cons.: percentage of consumption

Y_x/s_: Biomass yield coefficient

According to Wang et al., the level of tolerance of *C*. *tropicalis* to concentrated corn cob hydrolysate gradually increased with the process of adaptation [6]. Misra et al. stated that improvement in fermentation performance in the adapted strain may result from the ability of yeast to maintain the initial pH value, which in turn leads to the inactivation of toxic compounds present in the hydrolysate [3].

**Fig 3:**
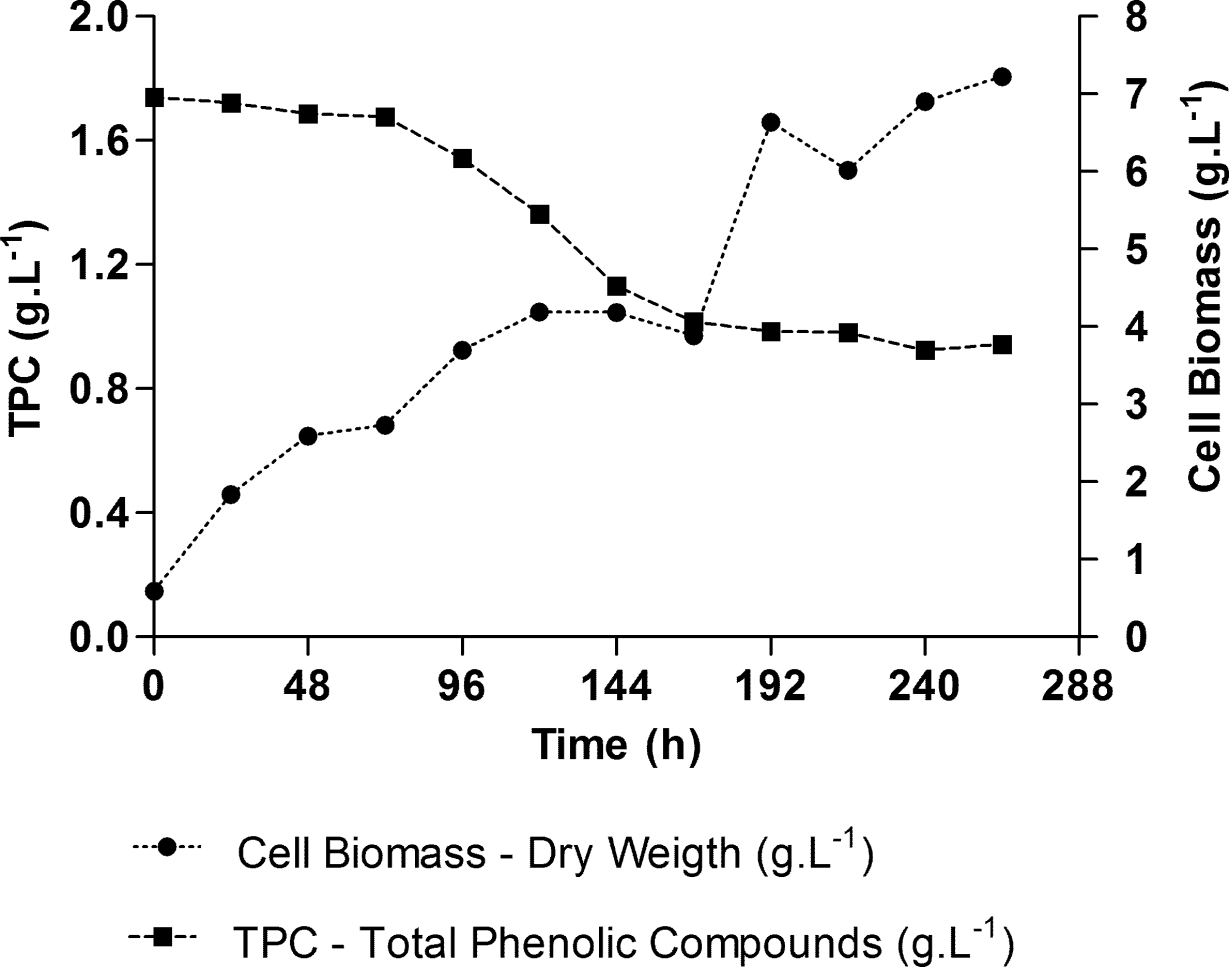
Cell growth monitoring and TPC decreasing by *C*. *boidinii* in non-detoxified CPHHH.

Detoxification with *C*. *boidinii* in the original concentration of CPHHH proved to provide the most favorable conditions of those tested for the reduction of such hemicellulose hydrolysate inhibitor compounds. Thus, this treatment was accompanied for a period of 264 h, in order to assess how cell growth is influenced by the presence of these compounds. The data are shown in Fig 3.

Phenolic compounds usually lead to a prolonged lag phase in the cells, and growth is only evident after reduction in the concentration of these compounds [39]. However, cell growth of *C*. *boidinii* was not as strongly inhibited by the presence of phenolic compounds present in the CPHHH without detoxification. Stabilization of cellular growth occurred between 120 h and 168 h of fermentation. When the TPC concentration reached approximately 1.0 g.L^-1^, the number of cells increased again after 168 h of growth, even though this concentration is relatively high and considered inhibitory to yeast. This may indicate a perfect adaptation of cells to TPC concentrations of approximately 1.0 g.L^-1^ or it could also be attributed to a phenomenon of diauxic growth, wherein another carbon source begins to be metabolized at this point, causing increased cell mass in the medium.

The decrease in furfural and HMF concentration were by 99.5% and 99.2%, respectively during 264 h of cultivation. The initial concentrations amounted to 0.32 g.L^-1^ and 0.4 g.L^-1^for furfural and HMF, respectively. The presence of these compounds at such concentrations did not cause an extension of the lag phase, similar to the phenolic compounds, as shown in Fig 3. This effect was also observed by Huang et al. with *C*. *tropicalis* JH030 in Napier grass hydrolysate containing 1.2 and 0.5 g.L^-1^ of furfural and HMF, respectively[12]. Nevertheless, *C*. *tropicallis* and *C*. *guilliermondii* showed growth only after the concentration of these compounds decreased in the medium, suggesting that the duration of the lag phase depends on the concentration of furfural and HMF [10, 11, 40]. The growth inhibition caused by furfural and HMF is probably a result of their action on several key glycolytic enzymes. The inhibitory effect of these compounds was most remarkable in fermentations conducted with low levels of inoculum, leading to a decrease in the initial number of viable cells [10, 40]. Huang et al.reported that furfural concentrations greater than 1.0 g.L^-1^exertan inhibitory effect on *Picchia stipites* [12]. However, the data obtained in this study show that the *C*. *boidinii* isolated presented high tolerance to these inhibitors, reducing the concentration of these compounds to similar levels to those obtained by traditionally applied physical and chemical methods of detoxification, as shown in Table 3.

In spite of the good adaptation of *C*. *boidinii* to the medium without detoxification, the ability to produce xylitol in fermentation medium was completely suppressed in the experimental conditions tested here. Wang et al. studied the effect of inhibitory compounds in *C*. *tropicallis* [10]. According to the authors, both the xylose uptake rate (XUR) and the xylitol oxidation rate (XOR) were stimulated in the groups treated with inhibitors. However, the XOR/XUR ratio was higher for the group treated with inhibitors than control group. This study has also observed an increase in the rate of consumption of xylose by pentose phosphate, which promotes the oxidation of xylitol, thus reducing its accumulation. The increased NADH/NAD+ ratio of the treated groups compared to the control also indicates that the oxidation of xylitol was enhanced. This may explain the fact that *C*. *boidinii* did not show accumulation of xylitol in medium without detoxification in the present work.

#### Fermentation of the CPHHH detoxified by pH adjustment and activated charcoal

**Fig 4:**
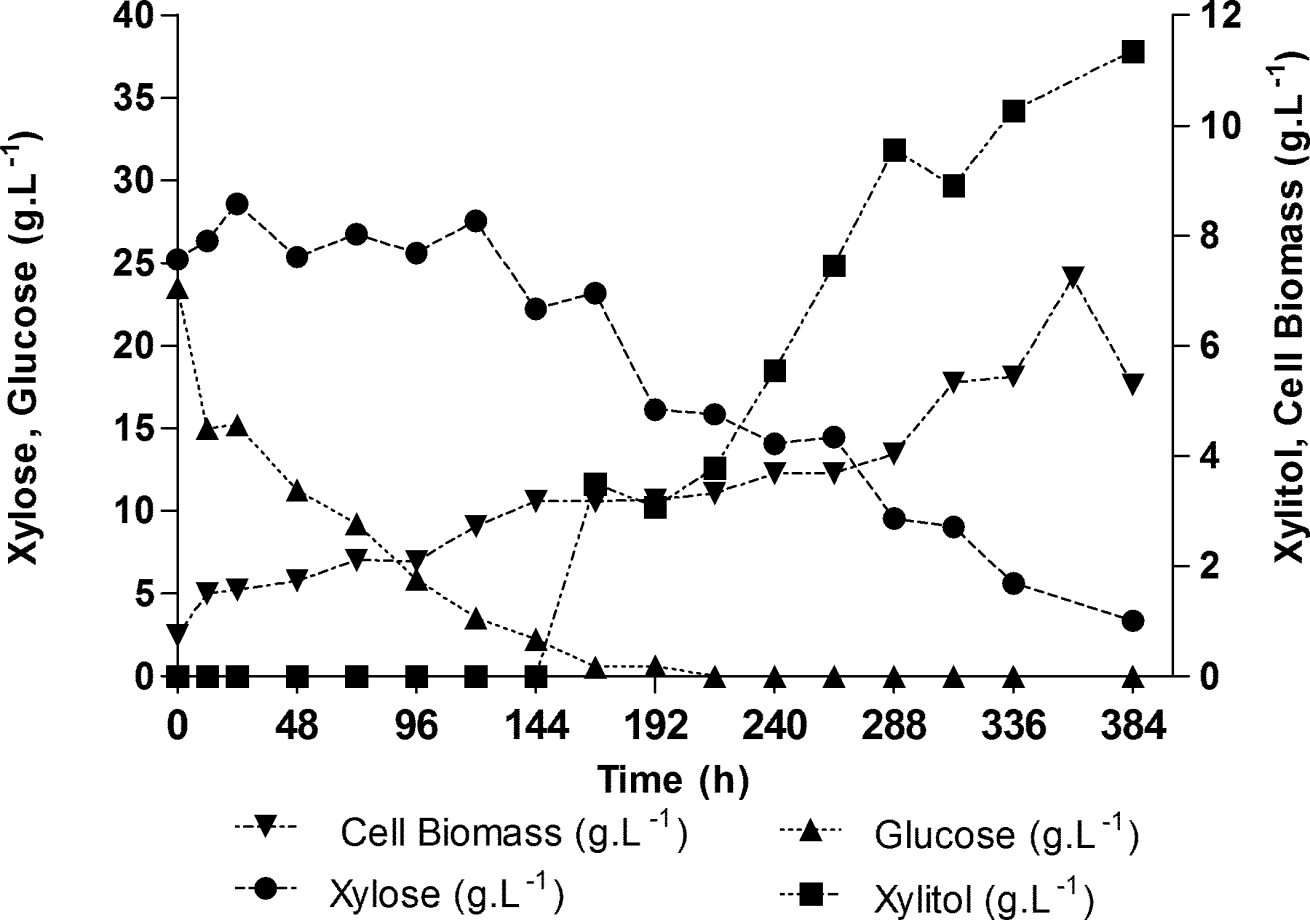
Fermentation of detoxified CPHHH by isolated *C*. *boidinii*.

The production of xylitol was studied in CPHHH detoxified by pH adjustment and activated charcoal, because this method is cheaper and has demonstrated similar efficiency to that of ion-exchange resins, and because there is still a lack of studies on biodetoxification. Fig 4 shows that due to the presence of large amounts of glucose in the CPHHH, xylose consumption effectively began after 120 h of cultivation, from which point there was a decrease in glucose levels. The production of xylitol also starts began after this period.

The presence of glucose causes suppression in the consumption of other carbon sources present in the growth medium. In the case of CPHHH fermentation, this amount of glucose can be used in the production of any other compound of interest, and the production of xylitol begins as soon as the sugar content starts to decrease. In this study, the xylose:glucose ratio was approximately 1:1 (w/w). Other studies indicate that glucose has a positive effect on the metabolism of xylose, and suggest that, in order to increase the production of xylitol, the optimal glucose:xylose ratio should range between 1:5 and 1:10 [12]. Cellular growth can be considered as moderate, in light of the large amount of substrate available in the medium. The final value for biomass produced was 5.29 g.L^-1^. Excessive production of biomass may indicate that the process is following an inappropriate path, providing conditions that favor higher biomass production at the expense of product formation. The final yield (Y_p/s_) in the production of xylitol was 0.52 g.g^-1^ with an efficiency (ε) of 56.6% in comparison to the stoichiometric yield. The highest value observed for Y_p/s_ was 0.69 g.g^-1^, obtained after 264 h and corresponding to an efficiency level ε of 75.56%. In Table 5 it is possible to compare the results obtained in this work with others found in the literature. The final consumption of xylose was 86.86% and the highest value achieved for xylitol was 11.34 g.L^-1^ after 372 h of fermentation. The xylitol concentration did not decrease even with the long fermentation time. Gonçalves et al. obtained xylitol values between 2 and 4 g.L^-1^ in fermentations with *C*. *boidinii* in hydrolysate of *macaúba pie*[41]. Vandeska et al. achieved xylitol values of 39.41 g.L^-1^ with *C*. *boidinii* in synthetic medium containing glucose and xylose, while in medium containing only xylose, the obtained values were 46.5 and 59.3 g.L^-1^[42]. The authors reported values of between 0.57 g.g^-1^ and 0.68 g.g^-1^ in fed batches.

**Table 5:** Concentration and yield of xylitol found in the literature.

Microorganism	Residue/medium	Xylitol(g.L^-1^)	Yield (g.g^-1^)	Reference
C. boidinii	cocoa pod husk	11.34	0.52	This work
*C*. *boidinii*	macaúba pie	2.0	n.i*	Gonçalves et al. [44]
*C*. *boidinii*	Sintetic medium	58.2	0.48	Vandeska et al. [42]
*C*. *tropicalis*	corncob	12.23	0.61	Misraet al [3]
*K*. *marxianus*	cashew apple	6.76	n.i	Rocha et al [35]
*C*. *tropicalis*	corn straw	35.6	0.71	Wang et al [40]
*C*. *tropicalis*	corncob	59.5	0.77	Jia et al [43]
*C*. *tropicalis*	corncob	12.2	0.61	Ping et al [11]
*C*. *guilliermondii*	eucalyptus	8.0	0.15	Villarreal et al [28]

*n.i: not informed

The maximum yield for xylitol of 0.72 g.g^-1^ for *C*. *tropicallis* was obtained when using concentrated corn cob hydrolysate twice as the medium. These authors postulated that the higher yield could be attributed to the rapid increase of yeast biomass, resulting from rapid glucose utilization during the initial stage of fermentation of the hydrolysates [39]. The yield for xylitol is not always negatively affected by hydrolysates. Huang et al. also noted that the yield obtained with the fermentation of xylitol from rice straw hydrolysate was greater than that obtained with the synthetic fermentation of xylose[12].

The long fermentation time obtained in this paper indicates the need for an adequate initial concentration of cells. Higher initial values of inoculum lead to shorter fermentation times and less inhibitory effects of toxic compounds. Wang et al. reported that the increase in the initial inoculum concentration from 0.4 g.L^-1^ to 1.6 g.L^-1^was followed by a 37% decrease in fermentation time [39]. Yield and productivity of xylitol respectively increased by 6% and 67.9%.

## Conclusions

The biotechnological reutilization of residual biomass is considered as an alternative for reducing environmental impacts and producing high value-added molecules.

*Candida boidinii* XM02G, isolated from cocoa cultivation areas, has shown interesting characteristics relating to the biotechnological production of xylitol because it proved able to withstand the toxicity present in CPHHH, even without any type of detoxification. This feature is interesting because it can result in reduction of steps and costs for the use of this biomass. The yield and efficiency values for the fermentation process reported in this study are interesting and in agreement with other published works.

These findings reinforce the importance of biodiversity for this region, since micro-organisms with differentiated characteristics can be found in this region. The use of the biomass of the cocoa pod husks to produce xylitol is an interesting proposal for reuse of this residue, which is abundant in the southern region of Bahiaand elsewhere in the world.

## Supporting information

S1 Supporting Information Subjacent data—Plos One. (DOCX)

S1 Fig Fermentation chromatogram of CPHHH. (TIF)
