# Supplementary material for: Production of xylitol and bio-detoxification of cocoa pod husk hemicellulose hydrolysate by Candida boidinii XM02G
Source: PLoS One. 2018 Apr 11;13(4):e0195206. doi: 10.1371/journal.pone.0195206 (PMC5895003; doi:10.1371/journal.pone.0195206)
Supplement: S1 Supporting Information — (DOCX) [file pone.0195206.s001.docx]

S1 Supporting Information. Subjacent Data

S1 Table - Result of Statistical Planning of hydrolysis

| Acid | Time | Xylose |
| --- | --- | --- |
| -1 | -1 | 2,29064358 |
| 1 | -1 | 11,0348175 |
| -1 | 1 | 5,09608494 |
| 1 | 1 | 9,71700183 |
| 0 | 0 | 10,0419421 |
| 0 | 0 | 8,79010194 |
| 0 | 0 | 10,8935892 |
| -1,41 | 0 | 0 |
| 1,41 | 0 | 9,64845009 |
| 0 | -1,41 | 6,16580329 |
| 0 | 1,41 | 10,4077898 |

S2 Table - Anova table for hydrolysis of the cocoa pod husk

|  | SS | df | MS | F | p |
| --- | --- | --- | --- | --- | --- |
| 1 Acid Linear | 91,1899 | 1 | 91,18989 | 81,45512 | 0,012055 |
| Acid Quad | 33,1008 | 1 | 33,10078 | 29,56718 | 0,032197 |
| 2 Time Linear | 6,9937 | 1 | 6,99373 | 6,24713 | 0,129660 |
| Time Quad | 2,6608 | 1 | 2,66075 | 2,37671 | 0,263090 |
| 1L by 2L | 4,2503 | 1 | 4,25031 | 3,79658 | 0,190698 |
| Lack of fit | 3,0650 | 3 | 1,02166 | 0,91259 | 0,560726 |
| Pure Error | 2,2390 | 2 | 1,11951 |  |  |
| Total SS | 140,8409 | 10 |  |  |  |

S3 Table - Composition of CPHH before and after detoxification.

| trat | rep | xilose | arab | glicose | CFT | FUR | HMF |
| --- | --- | --- | --- | --- | --- | --- | --- |
| NAT | 1 | 8,085932 | 3,61468 | 5,091369 | 0,71746 | 0,189999 | 0,41405 |
|  | 2 | 5,91983 | 2,293447 | 3,868517 | 0,973016 | 0,476826 | 0,415041 |
|  | 3 | 7,724893 | 2,002418 | 5,72291 | 1,047619 | 0,333413 | 0,414545 |
| RES 410 | 1 | 7,970962 | 1,789186 | 4,577544 | 0,284127 | 0,085601 | 0,209437 |
|  | 2 | 8,73315 | 1,63184 | 4,661376 | 0,226984 | 0,082374 | 0,055429 |
|  | 3 | 7,282312 | 1,194179 | 4,751598 | 0,295238 | 0,083987 | 0,132433 |
| RES 120 | 1 | 8,298614 | 0 | 2,005622 | 0 | 0,032666 | 0,025278 |
|  | 2 | 8,178788 | 0 | 3,098278 | 0,01746 | 0,027144 | 0,051571 |
|  | 3 | 8,826109 | 0 | 3,688299 | 0,02381 | 0,029905 | 0,038425 |
| PH 10 | 1 | 6,450669 | 3,085142 | 3,963011 | 0,428571 | 0,028525 | 0,044998 |
|  | 2 | 3,997764 | 1,769953 | 2,541434 | 0,579365 | 0,042407 | 0,04252 |
|  | 3 | 5,224217 | 2,427547 | 3,252222 | 0,54127 | 0,010373 | 0,007587 |
| PH 5 | 1 | 9,188379 | 2,698587 | 4,405657 | 1,039683 | 0,145726 | 0,149458 |
|  | 2 | 7,666684 | 4,250074 | 5,309418 | 1,019048 | 0,07805 | 0,078522 |
|  | 3 | 6,88118 | 3,778683 | 5,083524 | 0,911111 | 0,227605 | 0,302098 |
| PH 5,5 | 1 | 7,532109 | 1,806336 | 4,836742 | 0,565079 | 0,152827 | 0,19031 |
|  | 2 | 6,684921 | 3,858056 | 6,125333 | 0,761905 | 0,071958 | 0,150869 |
|  | 3 | 8,752658 | 2,092835 | 5,795308 | 0,78254 | 0,025536 | 0,015409 |
| DETOX 1X | 1 | 7,825136 | 1,333367 | 4,283845 | 0,093651 | 0,003919 | 0,003071 |
|  | 2 | 10,49042 | 1,731137 | 5,076302 | 0,193651 | 0,014727 | 0,00924 |
|  | 3 | 5,476316 | 2,421432 | 3,370194 | 0,169841 | 0,004797 | 0,004049 |
| DETOX 2X | 1 | 16,9254 | 0 | 6,563055 | 0,17619 | 0,009762 | 0,006644 |
|  | 2 | 19,20923 | 0 | 8,242954 | 0,363492 | 0,005498 | 0,011628 |
|  | 3 | 9,972606 | 6,931981 | 6,456487 | 0,322222 | 0,000918 | 0,003198 |
| DETOX 4X | 1 | 34,32181 | 10,04212 | 19,94866 | 0,366667 | 0,008459 | 0,012531 |
|  | 2 | 36,26071 | 10,24742 | 27,50827 | 0,542857 | 0,011898 | 0,018175 |
|  | 3 | 28,81503 | 13,91453 | 31,12913 | 0,674603 | 0,010178 | 0,015353 |

S4 Table - Original data for construction of figure 4.

| TEMPO | Biomass 1 (g/L) | Biomass 2 (g/L) | Biomass media | Gluc 1 | Gluc 2 | Gluc 3 | Media gluc | Media XILose G/L | Xylito media  g/l |
| --- | --- | --- | --- | --- | --- | --- | --- | --- | --- |
| 0 | 0,738 | 0,738 | 0,738 | 13,33333 | 12,47312 | 19,23203 | 15,01283 | 25,22537 | 0 |
| 12 | 1,488744 | 1,514161 | 1,501452 | 12,75986 | 12,47312 | 20,33712 | 15,19003 | 26,35259 | 0 |
| 24 | 1,6122 | 1,528686 | 1,570443 | 10,32258 | 9,892473 | 13,63993 | 11,28499 | 28,58765 | 0 |
| 48 | 1,761075 | 1,699346 | 1,730211 | 8,74552 | 9,032258 | 9,911471 | 9,229749 | 25,34946 | 0 |
| 72 | 2,196805 | 2,033406 | 2,115105 | 5,304659 | 4,874552 | 7,491445 | 5,890219 | 26,75784 | 0 |
| 96 | 2,200436 | 1,97894 | 2,089688 | 0 | 1,146953 | 5,925397 | 3,536175 | 25,60733 | 0 |
| 120 | 2,792302 | 2,657952 | 2,725127 | 1,72043 | 1,003584 | 4,028343 | 2,250786 | 27,55848 | 0 |
| 144 | 3,300654 | 3,057371 | 3,179012 | 1,290323 | 0,430108 | 0 | 0,573477 | 22,21938 | 0 |
| 168 | 3,169935 | 3,169935 | 3,169935 | 0,860215 | 1,003584 | 0 | 0,621266 | 23,18555 | 3,49634 |
| 192 | 3,238925 | 3,162672 | 3,200799 | 0 | 0 | 0 | 0 | 16,12009 | 3,081011 |
| 216 | 3,177197 | 3,471314 | 3,324256 | 0 | 0 | 0 | 0 | 15,84599 | 3,779273 |
| 240 | 3,652869 | 3,703704 | 3,678286 | 0 | 0 | 0 | 0 | 14,08237 | 5,548671 |
| 264 | 3,554829 | 3,82716 | 3,690995 | 0 | 0 | 0 | 0 | 14,46926 | 7,452971 |
| 288 | 4,030501 | 4,041394 | 4,035948 | 0 | 0 | 0 | 0 | 9,547638 | 9,554708 |
| 312 | 6,216412 | 4,444444 | 5,330428 | 0 | 0 | 0 | 0 | 9,036634 | 8,904916 |
| 336 | 6,071169 | 4,818446 | 5,444808 | 0 | 0 | 0 | 0 | 5,633567 | 10,27001 |
| 360 | 7,487291 | 6,964415 | 7,225853 | 0 | 0 | 0 |  | 3,37399 | 11,34121 |
| 384 | 6,652142 | 3,936093 | 5,294118 |  |  |  |  |  |  |

S5 Table - Original data of figure 3.

| Time | TPC r1 | TPC r2 | Biomass r1 | Biomass r2 |
| --- | --- | --- | --- | --- |
| 0 | 1,72238 | 1,9764 | 1,5565 | 1,6234 |
| 24 | 1,721133 | 1,94626 | 1,94626 | 1,833696 |
| 48 | 2,356572 | 2,81772 | 2,81772 | 2,587146 |
| 72 | 2,730574 | 2,723312 | 2,723312 | 2,726943 |
| 96 | 3,362382 | 4,02687 | 4,02687 | 3,694626 |
| 120 | 4,241104 | 4,139434 | 4,139434 | 4,190269 |
| 144 | 4,208424 | 4,157589 | 4,157589 | 4,183007 |
| 168 | 3,888889 | 3,881627 | 3,881627 | 3,885258 |
| 192 | 7,116921 | 6,158315 | 6,158315 | 6,637618 |
| 216 | 6,833696 | 5,19971 | 5,19971 | 6,016703 |
| 240 | 6,594045 | 7,218591 | 7,218591 | 6,906318 |
| 264 | 6,180102 | 8,271605 | 8,271605 | 7,225853 |

S6 Table - Original data for table 4.

| TRAT | REP | Conc inicial | Conc final |
| --- | --- | --- | --- |
| 1x R1 | 1 | 2,514286 | 1,638095 |
| 1X R2 | 2 | 1,879365 | 1,669841 |
| 1XR3 | 3 | 2,069841 | 1,84127 |
| 2X R1 | 1 | 2,812698 | 3,155556 |
| 2X R2 | 2 | 4,234921 | 3,650794 |
| 2X R3 | 3 | 3,530159 | 3,409524 |
| 3X R1 | 1 | 4,863492 | 4,336508 |
| 3XR2 | 2 | 4,780952 | 4,844444 |
| 4X ÚNICO | 1 | 5,92381 |  |
| 4X 96 R1 | 1 | 5,92381 | 5,980952 |
| 4X 96 R2 | 2 | 5,92381 | 5,771429 |
| 4X 120 R1 | 1 | 5,92381 | 6,209524 |
| 4X 120 R2 | 2 | 5,92381 | 5,263492 |
| 4X 20-10 R1 | 1 | 5,92381 | 6,025397 |
| 4X 20-10 R2 | 2 | 5,92381 | 4,761905 |

S7 Table - Original data for table 4 - Continued.

|  | Xylose inicial | Xylose final | %CONSUMO | biomass final | gluc in | | gluc fin | % consumo | delta S | delta X | Yx/s |
| --- | --- | --- | --- | --- | --- | --- | --- | --- | --- | --- | --- |
| 1x | 7,37 | 0,54 | 92,72 | 5,25 | | 5,493568 | 0,430967 | 92,15506 | 11,90 | 5,10 | 0,428782 |
| 2x | 12,02 | 4,60 | 61,70 | 3,83 | | 8,155585 | 0 | 100 | 15,57 | 3,80817 | 0,244574 |
| 3x | 17,52 | 11,39 | 34,98 | 2,95 | | 12,78407 | 0 | 100 | 18,91 | 2,933551 | 0,155128 |
| 4x (96) | 23,81 | 23,81 | 0 | 2,53 | | 17,67227 | 9,948221 | 43,70716 | 7,72 | 2,519735 | 0,326368 |
| 4X (20-10) | 23,81 | 21,32 | 0 | 2,77 | | 17,67227 | 0 | 100 | 20,17 | 2,759386 | 0,136838 |
| 4X (120) | 23,81 | 24,47 | 0 | 3,22 | | 17,67227 | 0 | 100 | 20,17 | 3,209641 | 0,159129 |
