# Supplementary figures and images for: Production of xylitol and bio-detoxification of cocoa pod husk hemicellulose hydrolysate by Candida boidinii XM02G
Source: PLoS One. 2018 Apr 11;13(4):e0195206. doi: 10.1371/journal.pone.0195206 (PMC5895003; doi:10.1371/journal.pone.0195206)

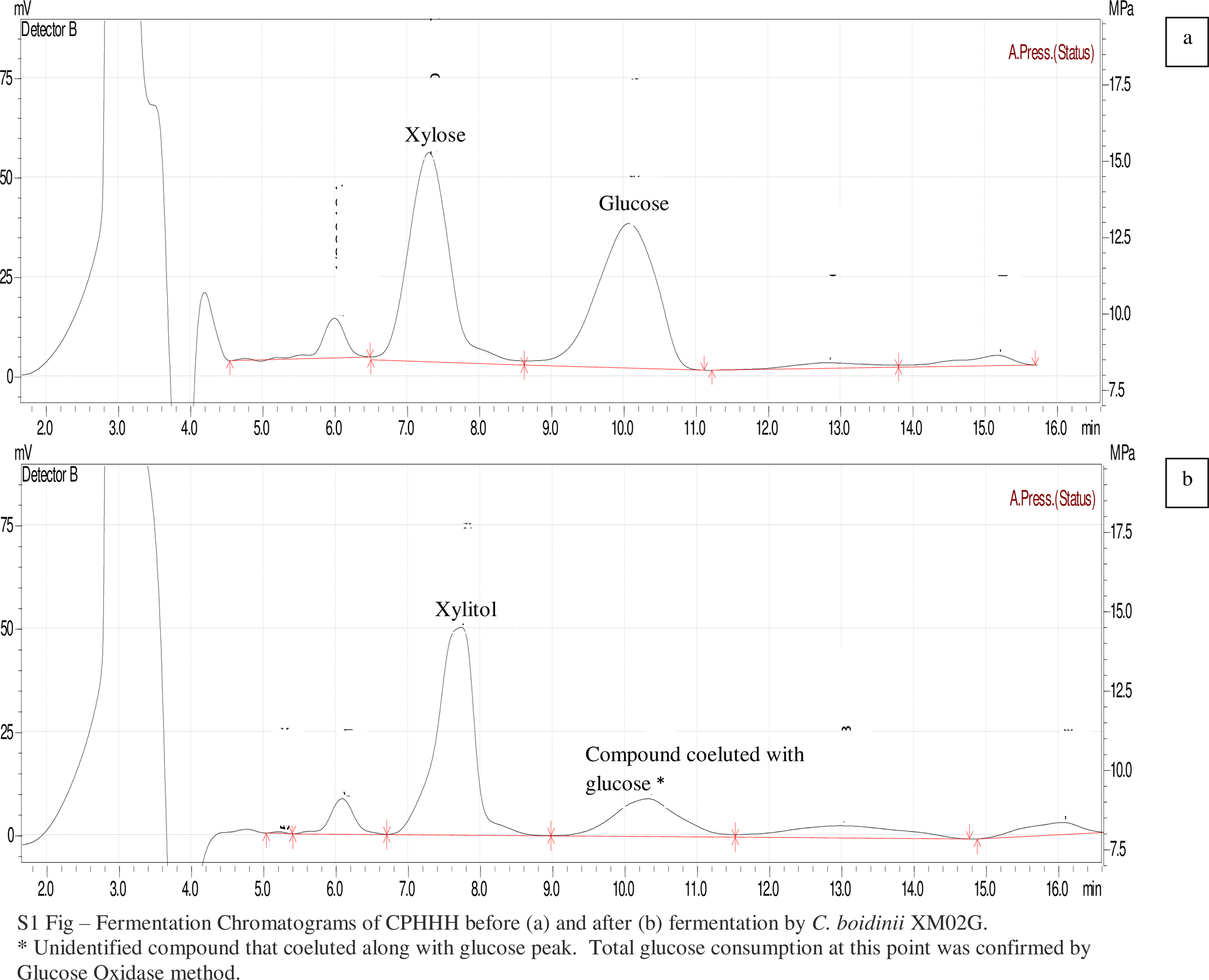

Supplement: S1 Fig — (TIF) [file pone.0195206.s002.tif]
